# Financial Incentives to Increase Diversity of Older Participants in a Memory Concerns Registry: A Randomized Clinical Trial

**DOI:** 10.1001/jamahealthforum.2025.2273

**Published:** 2025-08-22

**Authors:** Mireille Jacobson, Doris Molina-Henry, Tom Y. Chang, Gustavo A. Jimenez-Maggiora, Rajiv Pramanik, Samir B. Shah, Paul S. Aisen

**Affiliations:** 1 USC Schaeffer Institute, University of Southern California, Los Angeles; 2 Leonard Davis School of Gerontology, University of Southern California, Los Angeles; 3 Alzheimer’s Therapeutic Research Institute, University of Southern California, San Diego; 4 Department of Finance and Business Economics, University of Southern California, Los Angeles; 5 Contra Costa Regional Medical Center and Health Centers, Martinez, California

## Abstract

**Importance:**

Persons from marginalized racial and ethnic groups and of low socioeconomic status are at high risk of dementia but are underrepresented in clinical trials. Financial incentives may improve representation.

**Objective:**

To evaluate the effect of financial incentives on enrollment of county health system patients into a memory concerns registry.

**Design, Setting, and Participants:**

Between March 1, 2024, and April 24, 2024, patients 50 years and older without a dementia diagnosis within a single integrated county health system that includes a hospital and 9 outpatient health centers were invited to enroll in the Alzheimer Prevention Trials (APT) Webstudy, an online observational study aimed at accelerating enrollment into Alzheimer disease clinical trials.

**Interventions:**

Patients were randomized 1:1:1 to an invitation message (arm 1), a message with a small ($25) enrollment incentive (arm 2), or a message with an enrollment incentive of entry into a $2500 lottery with 1 in 100 odds of award (arm 3).

**Main Outcomes and Measures:**

The primary outcome was enrollment, defined as APT Webstudy registration and completion of at least 1 of 2 remote cognitive assessments. Outcomes were measured through April 30, 2024.

**Results:**

Of 44 844 patients invited to the APT Webstudy, the mean (SD) age was 64.7 (10.1) years, 25 447 (56.8%) were women, 25 044 (55.8%) had Medicaid insurance, 11 347 (25.3%) were Hispanic/Latino, 9526 (21.2%) were non-Hispanic Asian, 6044 (13.5%) were non-Hispanic Black, and 12 109 (27%) were non-Hispanic White. A total of 401 participants (0.9%) enrolled in the APT Webstudy. Relative to the message-only arm, participants randomized to the small incentive arm were more likely to enroll (adjusted odds ratio [OR], 1.39; 95% CI, 1.09-1.76; *P* = .008) in the APT Webstudy while those in the prize incentive arm were not more likely to enroll (adjusted OR, 1.08; 95% CI, 0.84-1.39; *P* > .99). Enrollment in the prize incentive arm was lower relative to the small incentive arm (adjusted OR, 0.78; 95% CI, 0.61-0.98; *P* = .04). Secondary heterogeneity analyses indicated that patients of White race (adjusted OR, 1.61; 95% CI, 1.15-2.25; *P* = .006) and male sex (adjusted OR, 2.40; 95% CI, 1.55-3.75; *P* < .001) were most responsive to the small $25 incentive relative to the message-only arm.

**Conclusions and Relevance:**

In this randomized clinical trial, relative to message invitations, invitations with guaranteed, small financial incentives but not lottery incentives increased enrollment of economically but not necessarily racially or ethnically diverse participants to a study that aimed to increase enrollment in clinical studies.

**Trial Registration:**

ClinicalTrials.gov Identifier: NCT06033066

## Key Points

**Question:**

Can financial incentives increase enrollment of primarily low-income, racially and ethnically diverse patients from a county health system into a memory concerns registry?

**Findings:**

In this randomized clinical trial including 44 844 adults 50 years and older, relative to an invitation message alone, a message with the offer of a $25 financial incentive increased enrollment, while a message with a $2500 prize incentive with 1 in 100 odds of award did not affect enrollment into the memory concerns registry.

**Meaning:**

Small financial incentives can increase enrollment of low-income patients into clinical research studies.

## Teaser

This randomized clinical trial evaluates the effect of financial incentives on enrollment of county health system patients into a memory concerns registry.

## Introduction

Women, older adults, Black and Hispanic/Latino(a) persons, and individuals with lower socioeconomic status are historically underrepresented in clinical trials.^1,2^ The past several decades have seen progress in clinical trial representation, but many marginalized groups continue to have low representation relative to their population and disease burden.^1^ Adequate clinical trial representation is important for multiple reasons, including strengthening the generalizability of findings, building trust in clinical trials and subsequent medical care, and promoting equitable access to the benefits (and costs) of novel clinical research.^3^ The latter 2 goals are arguably of highest social value given that powering trials to generalize across subgroups can be impractical and risks slowing scientific progress.^3,4^

Black and Hispanic older adults are underrepresented in Alzheimer disease (AD) clinical trials.^4,5,6,7,8^ This underrepresentation is noteworthy given their increased risk of AD and related dementias.^5,7,9,10,11,12,13,14,15^ Underrepresentation in clinical trials for recent novel AD therapeutics, which can alter the disease course for appropriate patients, was particularly severe for Black patients.^16,17,18,19,20^ This limitation may impact subsequent use of novel AD therapeutics and exacerbate disparities in treatment and outcomes given research suggesting Black patients are more likely to trust medications and physicians more willing to prescribe to Black patients when medications are tested in representative samples.^21^ The projected increase in dementia among Black and Hispanic older adults further increases the importance of improving clinical trial representation.^22^

Reasons for underrepresentation in clinical trials are manifold and include differential access to care and delays in diagnosis, differential awareness and knowledge of clinical studies, differential trust in clinical research studies, differential access to clinical trial sites, and differential impact of trial inclusion and exclusion criteria.^1,4,16,23,24,25,26^ While recruitment challenges are common across many disease areas, AD trials, which increasingly focus on early or even asymptomatic disease, face unique barriers, including a lengthy and costly screening process, high screen failure rates, long follow-up times to establish changes in disease progression, and study partner or caregiver participation requirements.^16,26,27,28^

The US Food and Drug Administration (FDA) draft guidance on diversity action plans for phase 3 clinical trials recommends several strategies for meeting enrollment goals, including increasing community engagement efforts, limiting study exclusion criteria, decentralizing study sites, and reimbursing participants for costs incurred.^29^ Using payment participation incentives is not discussed in the draft guidance. Earlier FDA guidance on payment incentives stated that paying research participants is “a common and, in general, acceptable practice”^30^ for recruiting. However, the FDA advises attention to the amount, timing, and method of payments to limit the potential for coercion or undue influence.^30,31^ Prior work has debated what constitutes reasonable and ethical payment incentives and demonstrates considerable variation in payment practices but has not generated clear guidelines for researchers.^32,33,34,35,36,37,38^

To add to evidence on the potential for financial incentives to increase diverse patient enrollment into clinical research, we conducted a stratified randomized clinical trial comparing the impact of email or text invitations with different financial incentives (small certain incentive or lottery incentive) relative to an invitation message alone on enrollment to a memory concerns registry that refers to AD clinical trials when appropriate. We also provide a rough estimate comparing the return on investment for each recruitment approach.

## Methods

### Study Design

The study used a single-blind, 3-arm parallel intention-to-treat design to compare the effect of invitation messages with a certain small financial incentive or a lottery incentive with an invitation without an incentive on enrollment into the Alzheimer Prevention Trials (APT) Webstudy, an online observational study of adults 50 years and older who are not already diagnosed with dementia and who are routinely assessed through the platform for eligibility and recruitment into early-stage AD trials.^39,40,41^ Initial enrollment involves an online consent process, completion of some demographic and family history questions, and assessment via the Cogstate Brief Battery, which measures cognitive performance, and/or the Cognitive Function Index (CFI), which captures subjective memory concerns related to cognitive performance.^42,43^ Enrolling and completing the CFI takes about 10 minutes; enrolling and completing both assessments takes about 30 minutes.

Invitation messages were sent to adult patients 50 years and older without documentation of dementia from the Contra Costa Regional Medical Center (CCRMC), an integrated county health system that includes a hospital and 9 outpatient health centers. Most health system patients are enrolled in Medicaid. The intervention was deployed March 1 to April 16, 2024, with data collected through April 30, 2024.

Both the University of Southern California Institutional Review Board and Contra Costa Health Institutional Review Committee approved this study and provided a waiver of patient consent because the trial was low risk as it simply encouraged patients to enroll in a registry and it was impractical to obtain consent from 45 000 patients. Individuals who enrolled in the APT Webstudy provided consent and Health Insurance Portability and Accountability Act (HIPAA) authorization through that study’s separate approved protocol. Study data were deidentified. The trial protocol is in Supplement 1, and a study overview can be found in eAppendix 1 in Supplement 2. This study followed the Consolidated Standards of Reporting Trials (CONSORT) reporting guideline.

### Study Participants

Eligible patients were empaneled at CCRMC, 50 years or older, had not opted out of health system messaging, and did not have a dementia diagnosis documented in the electronic medical record.

### Randomization and Blinding

Patients were allocated 1:1:1 to study arms. The Biostatistics team at the University of Southern California Alzheimer Therapeutic Research Institute generated the allocation sequence using a stratified randomization sampling method. Strata were based on age group (aged 50 to 64 years or 65 years and older ), race and ethnicity (Asian, Black, Hispanic/Latino[a], White, or other race), and message modality (email or text). The research team was blinded to study arm assignment.

Contact information and demographic data were from the electronic medical record, with race and ethnicity self-reported. Patients were classified as Hispanic/Latino(a) regardless of race. Due to small sample sizes, patients who self-identified as American Indian or Alaska Native, Native Hawaiian or Other Pacific Islander, or multiracial were grouped with those of other or unknown race in the other race category.

### Intervention

**Figure 1.**
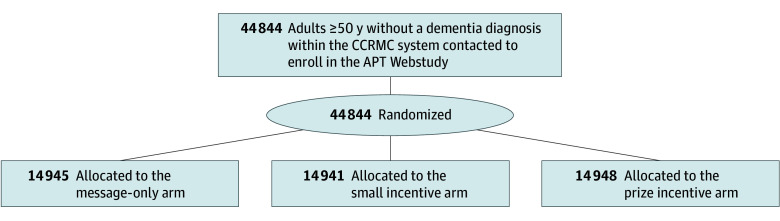
Study Flow Diagram All randomized patients were included in the final analysis. APT indicates Alzheimer Prevention Trials; CCRMC, Contra Costa Regional Medical Center.

Participants were randomized to 1 of 3 possible arms: (1) a message-only group that was sent a text or email message by the health system inviting participants to enroll in the APT Webstudy (n = 14 945), (2) a small financial incentive arm that was sent the same message but with the offer of a $25 Amazon gift card incentive for enrolling in the APT Webstudy within 1 week of the message (n = 14 951), or (3) a prize incentive arm that was sent the same message but with the offer of entry into a prize drawing for $2500 Amazon gift card with a 1-in-100 odds of award for enrolling within 1 week of the message (n = 14 951) (Figure 1). The simple invitation message served as an active control arm. Participants in all arms were sent 1 message via email or text, depending on preferred method of communication. All messages briefly explained the APT Webstudy and that participation may contribute to knowledge about dementia. Messages were sent through the health system’s standard email and text communication system in English or Spanish based on a participant’s language preference. Individuals who indicated a preferred language other than English or Spanish were sent the English message. Messages are provided in eAppendix 2 in Supplement 2. We hypothesized that financial incentives would increase enrollment relative to a message alone.

### Data and Measures

The primary outcome was APT Webstudy enrollment within 2 weeks of messaging as documented by the APT Webstudy team. The 2-week time frame was chosen based on work showing that shorter actionable windows are more effective in inducing action related to health care prevention.^44^ APT Webstudy enrollment requires completion of study consent and HIPAA authorization, and at least 1 of the 2 online cognitive assessments. We also prespecified 2 secondary outcomes—completion of both cognitive assessments and cost per enrollee.

APT Webstudy enrollment status was tracked via a unique URL embedded in each study invitation and shared by the APT Webstudy team. All other data, including participant age, race and ethnicity, sex, preferred mode of communication, and preferred language, came from CCRMC’s electronic medical record.

### Power Calculation

Power calculations were based on a 2-sided χ^2^ test for detecting a difference between 2 proportions, assuming a type I error rate of 2.5% (to account for the 2 incentive relative to control comparisons). We assumed a sample size of 11 000 participants per group (33 000 overall), and an enrollment rate of 2% in the active control arm, which would have given us 90% power to detect an absolute difference of 0.72% (an enrollment rate of 2.72%) in either of the 2 financial incentive groups. In practice, we had larger sample sizes (roughly 15 000 per arm) and a much lower control group enrollment rate of 0.78%. Assuming a type I error rate of 2.5% and standard power of 80%, we would be able to detect an absolute difference of 0.35% (an enrollment rate of 1.13%) in either of the 2 financial incentive groups.

### Statistical Analysis

As prespecified, our primary analysis focused on APT Webstudy enrollment comparing our intervention arms (small or prize incentive) relative to the message-only active control arm. The analysis used multivariate logistic regression models controlling for stratification variables—age group (aged 50 to 64 years or 65 years and older), race and ethnicity status (Asian, Black, Hispanic/Latino[a], White, or other race), and message modality (email or text messaging). In secondary analysis using the same model, we assessed whether enrollment differed across financial incentive arms (prize incentive relative to small incentive). We estimated robust standard errors and reported results as odds ratios (ORs) with 2-sided 95% CIs.

We also conducted secondary analyses that fit separate logistic regression models by subgroup. We considered subgroups defined by stratification variables and based on sex and insurance type. Subgroup models by stratification variables controlled for other stratification variables. For example, models by race and ethnicity controlled for age group and message type. We also analyzed completion of both cognitive assessments. Finally, we computed the per-arm cost of an additional enrollee to assess the return on investment in each arm. We assumed marginal costs of $0 per email and $0.05 per text message.^45^

For the primary analysis comparing each of the intervention arms to active control, statistical significance was set at 2-tailed *P* < .025 to address multiple hypothesis testing. Secondary analyses did not correct for multiple hypotheses and used 2-tailed *P* < .05. All *P* values were calculated using χ^2^ tests. All statistical analyses were performed using Stata version 18 (StataCorp).

## Results

### Participant Characteristics

A total of 44 844 participants (mean [SD] age, 64.7 [10.1] years) were included in the study (Figure 1). Of these, 9526 (21.2%) were Asian, 6044 (13.5%) were Black, 11 347 (25.3%) were Hispanic/Latino(a), 12 109 (27.2%) were White, and 5818 (13.0%) were another race (Table 1). Most participants (27 155 [60.6%]) were invited to the APT Webstudy by email, and 17 689 (39.4%) were invited by text. A total of 25 447 participants (56.7%) were female and 19 397 (43.3%) were male. As primary insurance, 25 044 (55.8%) had Medicaid, 12 590 (28.1%) Medicare, 2709 (6.9%) had commercial insurance, and 4521 (10.1%) had other coverage. Nearly 1 in 5 individuals (8813 [19.7%]) indicated that Spanish was their preferred language for communication with the health system.

**Table 1.** Baseline Characteristics by Study Arm

Characteristic	No. (%)			
	Message-only arm	Small incentive arm	Prize incentive arm	Overall
Age, mean (SD), y	64.7 (10.1)	64.7 (10.1)	64.6 (10.1)	64.7 (10.1)
Age group, y				
50-64	8180 (54.7)	8184 (54.7)	8182 (54.7)	24 546 (54.7)
≥65 y	6765 (45.3)	6767 (45.3)	6766 (45.3)	20 298 (45.3)
Race and ethnicity^a^				
Asian	3174 (21.2)	3176 (21.2)	3176 (21.3)	9526 (21.2)
Black	2014 (13.5)	2017 (13.5)	2013 (13.5)	6044 (13.5)
Hispanic/Latino(a)	3782 (25.3)	3782 (25.3)	3783 (25.3)	11 347 (25.3)
White	4036 (27.0)	4037 (27.0)	4036 (27.0)	12 109 (27.0)
Other race	1939 (13.0)	1939 (13.0)	1940 (13.0)	5818 (13.0)
Message modality				
Text messaging	5894 (39.4)	5898 (39.5)	5897 (39.5)	17 689 (39.5)
Email	9051 (60.6)	9053 (60.6)	9051 (60.6)	27 155 (60.6)
Sex				
Female	8490 (56.8)	8409 (56.2)	8548 (57.2)	25 447 (56.8)
Male	6455 (43.2)	6542 (43.8)	6400 (42.8)	19 397 (43.3)
Message language				
English	11 983 (80.2)	12 007 (80.3)	12 041 (80.6)	36 031 (80.4)
Spanish	2962 (19.8)	2944 (19.7)	2907 (19.5)	8813 (19.7)
Primary insurance				
Medicaid (Medi-Cal)	8317 (55.7)	8377 (56.0)	8330 (55.7)	25 024 (55.8)
Medicare	4194 (28.1)	4242 (28.4)	4154 (27.8)	12 590 (28.1)
Commercial	925 (6.2)	885 (5.9)	899 (6.0)	2709 (6.0)
Other	1509 (10.1)	1447 (9.7)	1565 (10.5)	4521 (10.1)

^a^ Race and ethnicity data were self-reported. Other race includes participants who identified as American Indian or Alaska Native, Native Hawaiian or Other Pacific Islander, multiracial, other race, or declined to provide or were otherwise missing race information.

### Primary Analysis

**Figure 2.**
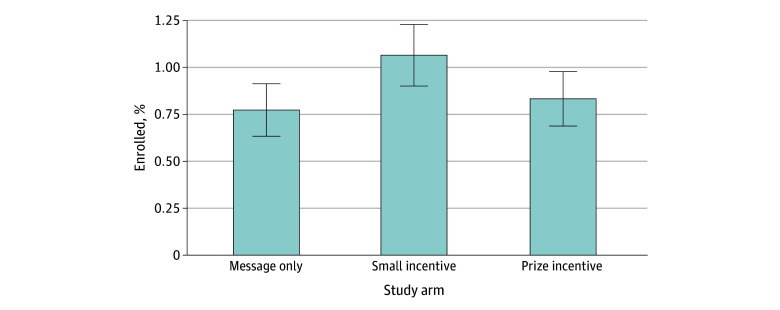
Enrollment Rates by Study Arm Enrollment in the small incentive arm (*P* = .008) but not the prize incentive arm (*P* > .99) was statistically different from the message only arm. Error bars indicate 95% CIs.

Figure 2 shows APT Webstudy enrollment rates by study arm. Enrollment was low. In the control arm, only 116 participants (0.8%) who were messaged enrolled, meaning they registered and completed at least 1 of 2 cognitive assessments (Figure 2). Relative to the control arm, enrollment was about 40% higher in the small incentive arm (160 [1.1%]; *P* = .008) but not statistically different in the prize incentive arm (125 [0.8%]; *P* > .99).

These basic patterns held in both the unadjusted and adjusted logistic regression models (Table 2). Odds of enrollment in the APT Webstudy were systematically higher in the small incentive arm (adjusted OR, 1.39; 95% CI, 1.09-1.76; *P* = .008). In contrast, the odds of enrollment were neither statistically different nor meaningfully different in magnitude in the prize incentive arm (OR, 1.08; 95% CI, 0.84-1.39; *P* = .56).

**Table 2.** Primary Outcome by Study Arm

Subgroup^a^	Enrollment rate in the message-only arm, No./total No. (%)	Unadjusted OR (95% CI)	*P* value^b^	Adjusted OR (95% CI)^c^	*P* value^b^
All participants					
Small incentive arm	116/14 945 (0.9)	1.38 (1.09-1.76)	.008	1.39 (1.09-1.76)	.008
Prize incentive arm		1.08 (0.84-1.39)	.56	1.08 (0.84-1.39)	.56
Asian					
Small incentive arm	8/3174 (0.3)	1.88 (0.80-4.44)	.15	1.88 (0.80-4.44)	.15
Prize incentive arm		1.38 (0.55-3.42)	.49	1.38 (0.55-3.43)	.49
Black					
Small incentive arm	16/2014 (0.8)	1.06 (0.54-2.11)	.87	1.06 (0.53-2.11)	.87
Prize incentive arm		0.938 (0.46-1.90)	.86	0.938 (0.46-1.90)	.86
Hispanic					
Small incentive arm	24/3782 (0.6)	1.00 (0.57-1.76)	>.99	1.00 (0.57-1.77)	>.99
Prize incentive arm		0.832 (0.46-1.51)	.55	0.832 (0.46-1.51)	.55
White					
Small incentive arm	56/4036 (1.4)	1.60 (1.14-2.25)	.006	1.61 (1.15-2.25)	.006
Prize incentive arm		1.09 (0.76-1.57)	.64	1.09 (0.76-1.57)	.64
Other race^d^					
Small incentive arm	12/1939 (0.6)	1.25 (0.58-2.68)	.56	1.25 (0.58-2.69)	.56
Prize incentive arm		1.50 (0.72-3.13)	.28	1.51 (0.72-3.15)	.27

Abbreviation: OR, odds ratio.

^a^ Estimates for subgroup analyses are from separate logistic regression models.

^b^ *P* values are from a test of the null that the odds of enrollment in a given arm is the same as the odds in the message-only (active control) arm.

^c^ Models for all participants adjusted for race and ethnicity (Asian, Black, Hispanic, or other race relative to White race), age group (65 years and older relative to younger than 65 years), and message modality (email relative to text). Models by race and ethnicity only controlled for age group and message modality.

^d^ Other race includes participants who identified as American Indian or Alaska Native, Native Hawaiian or Other Pacific Islander, multiracial, other race, or declined to provide or were otherwise missing race information.

### Secondary Analysis

We tested whether the prize incentive arm and small incentive arm impacts were the same (eTable 1 in Supplement 2). The odds of enrollment was lower in the prize arm relative to the small incentive arm (OR, 0.78; 95% CI, 0.62-0.99; *P* = .04).

**Figure 3.**
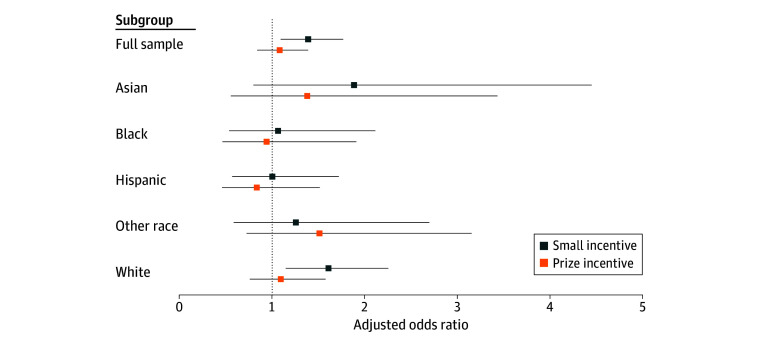
Odds Ratios From Models Estimated by Race and Ethnicity Groups The other race group includes American Indian or Alaska Native, Native Hawaiian or Other Pacific Islander, multiracial, other race, or declined to provide or were otherwise missing race information. Whiskers indicate 95% CIs.

We also estimated models by subgroup. ORs from logistics regression models estimated by race and ethnicity are reported in Table 2 and plotted in Figure 3. Only among White participants can we reject that the impact of the small incentive arm is the same as the message-only arm (adjusted OR, 1.61; 95% CI, 1.15-2.25; *P* = .006). For all other race and ethnicity groups, the small incentive arm was statistically indistinguishable from the message-only arm and, with the exception of participants who identify as Asian (OR, 1.88; 95% CI, 0.80-4.44; *P* = .15), the magnitude of the ORs was close to 1. Like the full-sample results, the impact of the prize incentive arm was never distinguishable from the message-only arm.

ORs for models by age group, message type, sex, and primary insurance are reported in eTable 2 and the eFigure in Supplement 2. The odds of enrollment in the small incentive arm was higher than in the message-only arm for participants across both message modalities (email: OR, 1.31; 95% CI, 1.01-1.70; *P* = .046; text: OR, 1.83; 95% CI, 1.01-3.31; *P* = .046) as well as for participants aged 50 to 64 years (OR, 1.52; 95% CI, 1.13-2.06; *P* = .06) but not for participants 65 years and older (OR, 1.16; 95% CI, 0.77-1.74; *P* = .47). Across sex and primary insurance subgroups, the most notable patterns were that the odds of enrollment in the small incentive arm was higher than in the message-only arm for male participants (OR, 2.41; 95% CI, 1.55-3.75; *P* < .001) and for participants with Medicaid as the primary payer (OR, 1.54; 95% CI, 1.11-2.13; *P* = .01). Across none of the subgroups was the prize incentive statistically distinguishable from the message-only arm.

eTable 3 in Supplement 2 shows ORs for completion of both cognitive screeners (CFI and CogState) for the full sample and by subgroups. Similar to the findings for enrollment, the odds of completion of both screeners was higher for those in the small incentive arm relative to the message-only arm (OR, 1.82; 95% CI, 1.18-2.80; *P* = .007), while the odds for the prize incentive arm was statistically indistinguishable from the message-only arm. The higher odds for the small incentive arm were most pronounced for male participants (OR, 3.41; 95% CI, 1.55-7.49; *P* = .002) and participants whose primary insurance was through Medicaid (OR, 3.39; 95% CI, 1.54-7.46; *P* = .002).

With 40% of messages sent by text and 0.78 enrolled per 100 messages sent, it cost $2.56 to recruit an additional person in the message-only arm (eTable 4 in Supplement 2). Recruitment costs increased to $26.87 in the small incentive arm and $27.38 in the prize incentive arm.

## Discussion

A January 2025 Executive Order to terminate policies related to diversity, equity, and inclusion led to the removal of the FDA’s Diversity Action Plan draft guidance on enrollment of underrepresented people in clinical studies, although a court order temporarily restored it.^46,47^ Nonetheless, achieving representativeness of at-risk populations in research remains a scientific and social imperative. Better representation can improve the generalizability of study fundings, contribute to understanding mechanisms of action, and increase public trust in medicine.^1,2,3,4^ Heart failure trials, including landmark trials sufficiently powered for subgroup analyses based on hypothesized differences in disease mechanisms, demonstrate that diverse representation can reduce morbidity and mortality.^48,49,50,51^

Rigorous evidence on how to cost-effectively improve representativeness is limited. To add to the evidence, we tested remote messaging with and without financial incentives to recruit a racially and ethnically diverse group of primarily low socioeconomic status patients from a county health system to an online observational study that serves as a connector to clinical trials.

Consistent with other patient text and email recruitment efforts, enrollment was low.^52,53,54,55^ The small $25 incentive increased enrollment by almost 40%, consistent with a pediatric trial registry study.^56^ The impact of the small incentive was clearest for participants who were White, male, or had Medicaid as the primary insurance. The male participant response was noteworthy given their underrepresentation in AD clinical trials based on population, albeit not disease prevalence.^57^ The lottery incentive did not increase enrollment, even though insights from behavioral economics suggest they can work better than small guaranteed payments with the same expected value.^58,59^ This finding is, however, consistent with a scoping review of financial incentives for trial recruitment and retention.^60^

Although the $25 incentive increased enrollment, the message alone was significantly less expensive, costing approximately $2.50 per additional enrollee vs $26.87 in the intervention arms. Furthermore, the 116 enrollees in the message-only arm were racially and ethnically diverse, with 16 Black participants (13.8%) and 24 Hispanic participants (20.7%) (eTable 5 in Supplement 2). Instead of financing incentives, registries might be better served using funds to build connections to and trust with underrepresented groups. Low-cost recruitment messages could serve to increase both the size and diversity of study participants.

### Limitations

Our study has several limitations. First, payments were made via Amazon gift cards, and not everyone has an Amazon account or values the cards equally. Although more than 90% of people in the US who shop online have used Amazon,^61^ Black and Hispanic/Latino(a) older adults may be less likely to have Amazon accounts or use the platform.^62,63^ As such payment form may have contributed to the null findings for Black and Hispanic participants. Second, due to telecommunications regulations, our messages could not include terms like “dollar” or the “$” symbol, making it harder to advertise incentives. Third, the campaign was limited to 1 message because of concerns about overmessaging. Multiple and clearer messages might have had bigger enrollment impacts. Finally, because we recruited to a registry, our findings may not generalize to recruitment to clinical intervention studies. Despite these limitations, our study had several strengths, including the use of a large, pragmatic randomized clinical trial in a low-income and racially and ethnically diverse health system, the comparison of messages to 2 different financial incentives, and the tracking of enrollment rather than intentions to enroll.

## Conclusions

In this large pragmatic randomized clinical trial in an integrated county health system, small financial incentives, but not lottery incentives, increased enrollment of economically diverse participants into a memory concerns registry. If researchers partner with diverse communities willing to receive invitations, however, a message alone is the more cost-effective and scalable approach to enrolling underrepresented participants.

## Notes

Supplement 1. Trial Protocol

Supplement 2. **eAppendix 1.** Study Overview **eAppendix 2.** Study Messages **eFigure.** Odds Ratios From Models Estimated by Subgroups **eTable 1.** Odds Ratios for APT Webstudy Enrollment Estimated Relative to the Small Incentive Arm **eTable 2.** Odds Ratio for Enrollment in the APT Webstudy by Age Group, Sex, and Primary Insurance **eTable 3.** Odds Ratio for Completion of Both Screeners Overall and by Subgroup **eTable 4.** Marginal Cost Per Additional Enrollee **eTable 5.** Characteristics of APT Webstudy Enrollees Overall and by Arm

Supplement 3. Data Sharing Statement
