# Supplementary material for: Financial Incentives to Increase Diversity of Older Participants in a Memory Concerns Registry: A Randomized Clinical Trial
Source: JAMA Health Forum. 2025 Aug 22;6(8):e252273. doi: 10.1001/jamahealthforum.2025.2273 (PMC12374222; doi:10.1001/jamahealthforum.2025.2273)
Supplement: Supplement 1. — Trial Protocol [file jamahealthforum-e252273-s001.pdf]

Study Title: A Randomized Trial of Financial Incentives for the Recruitment of County Health System Patients to the Alzheimer Prevention Trials (APT) Webstudy

PI Name: Mireille Jacobson

Study Procedures

1. Background/Rationale

Racial and ethnic disparities in Alzheimer's disease and related dementias (ADRD) are pervasive. Major racial and ethnic inequalities in dementia incidence have been reported across cohort studies [1]. Compared to Whites and Asians, Blacks and Hispanics have a higher prevalence of dementia [2, 3]. These differences carry into disease severity and presentation [4, 5]. African American/Black and Hispanic/Latino(a) patients display a greater severity of cognitive symptoms at time of disease onset [6].

Despite this level of vulnerability to ADRD and the increasing racial and ethnic diversity of the US population [7], Hispanic/Latino individuals and non-white individuals of lower socioeconomic status and lower educational levels are minimally represented in clinical trials [8, 9]. The FDA reports that only 5% of clinical trial participants are Black, and only 1% are Latino(a) [10]. These proportions grossly underrepresent the respective distributions of these groups within the US population [7]. The concerted efforts from guiding agencies and experts have helped to prioritize the increased representation of groups with diverse demographic attributes [11, 12]. Nevertheless, barriers to recruitment of underrepresented groups into clinical trials remain [13, 14].

AD clinical trials, especially those targeting the preclinical/prodromal stage, face specific hurdles. AD trials are frequently delayed due to slow patient enrollment, long follow-up periods, higher costs, and other operational challenges compared with trials in other therapeutic areas [16]. The challenges in enrollment are further exacerbated when it comes to populations of diverse demographic backgrounds, particularly non-white individuals who have markedly higher rates of AD but lower access to and use of care than non-Hispanic Whites [17-19]. The FDA indicates that less than 6% of clinical trial participants are from these underrepresented groups. With the projected increase of AD over the next three decades among individuals who identify as being African American/Black, Hispanic/Latino(a), and Asian, increasing representation of these groups across preclinical/prodromal AD clinical trials is critical. Implementation of inclusive recruitment strategies that engage these populations is of paramount importance.

The goal of this work is to explicitly test whether financial incentives can be used to increase the diversity of participants recruited to the Alzheimer Prevention Trials (APT) Webstudy (USC IRB, HS-17-00746), an innovative online registry of adults with concerns about memory problems who are routinely assessed for eligibility and recruitment into the Trial Ready Cohort for the Prevention of Alzheimer's Disease (TRC-PAD) (Advarra IRB, Pro00030367) and ultimate enrollment into early-stage ADRD trials [15].

The APT Webstudy is a longitudinal study that serves as a web-based registry to improve the efficiency at which participants at early stages of AD can be screened. Screening within the APT Webstudy occurs

through quarterly remote cognitive assessments. Data is analyzed monthly using an adaptive algorithm to assess each participant's risk of AD. Participants at high risk for AD are referred for cognitive testing, genotyping, and amyloid biomarker measures at clinical trial sites to determine Trial-Ready Cohort (TRC) eligibility. Participants that are TRC eligible are enrolled and followed longitudinally until an appropriate clinical trial becomes available. Thus, the APT Webstudy is an important first step in casting a wide net to identify individuals that may not yet have concerns related to their memory and may not have sought medical attention with cognitive concerns. The convenience of remote assessment and the low burden to potential clinical trial participants make it an excellent platform to evaluate recruitment and retention practices across populations. To date, the APT Webstudy has consented over 56,000 participants. The APT Webstudy and TRC-PAD Study are overseen by USC's Alzheimer's Therapeutic Research Institute (ATRI).

While remote registries, like the APT Webstudy, offer numerous advantages such as accessibility and low burden to individuals eligible for in-person multisite trials, particularly at the recruitment level, representation from diverse groups is still limited. A well-known barrier to ethnocultural diversity is the lack of trust in the academic and medical establishments that are often involved in clinical trials [3]. Approaching issues of mistrust requires time, relationship building and resources [13, 24, 25] that are not often built into the clinical trial infrastructure. Numerous studies show that collaboration with existing trusted community partners in recruitment may be effective at increasing the enrollment of minoritized communities into research [25-27].

Financial incentives are common in behavioral change interventions including medication and exercise adherence and hypertension management [28-31]. Results from a weight loss study indicate that participants who received financial incentives were more likely to temporarily maintain weight loss than those who did not [1]. In a tobacco cessation program, African American and English-speaking Latino(a) participants were more engaged when financial incentives were included [32]. An earlier RCT aimed at smoking cessation showed that in a diverse cohort of 352 underserved adults (mean age of 50) at a large urban safety-net hospital, patient navigation and financial incentives significantly increased rates of uptake. Subgroup analyses suggested that the navigation and financial incentives were particularly beneficial in older participants, women, non-Hispanic White and lower socioeconomic status (SES) participants [33]. Although participants are commonly compensated for their enrollment in clinical trials, as well as at the assessment of primary endpoints, financial incentives have seldom been used in AD trials. Notably, lottery systems have recently been adopted to incentivize individuals to receive the COVID-19 vaccination [34]. Research into understanding which method of incentivizing AD participants is most effective from a recruitment standpoint and from a cost standpoint would be valuable for all future trials.

2. Purpose/Objectives/Aims/Research Questions

<u>Primary Objectives:</u> To assess the impact of offering financial incentives (a certain small financial incentive or a randomized prize incentive) on enrollment rates of a population of diverse low-income adults ages 50 years and older into the APT Webstudy.

<u>Secondary Objectives</u>: To test whether the prize incentive is more effective at increasing enrollment than the certain small financial incentive. To assess the impact of financial incentives on cost per enrollee and, separately, completion of two cognitive assessments.

<u>Tertiary Objectives:</u> To analyze all primary and secondary outcomes for the following subgroup characteristics at baseline: sex (M/F), race and ethnic groups (Latino/Hispanic, non-Hispanic White, non-Hispanic Black, non-Hispanic Asian, Other), primary language (English/Spanish), message modality (email or text) and age group (50-64, 65-74, 75 and over).

3. Participants (sample)

Inclusion Criteria:

To be eligible to participate in this study, an individual must meet all of the following criteria:

i. Patients empaneled at Contra Costa Regional Medical Center (CCRMC), the county public hospital and its affiliated health centers.
ii. Age 50 or older
iii. Literate in either English or Spanish

Inclusion criteria are justified because only those age 50 and over are eligible for the APT Webstudy and the APT Webstudy is only available in English and Spanish. Because CCRMC is a county health system, most of its patients are low income. They also are racially and ethnically diverse. These characteristics are under-represented in brain health registries and clinical trials for AD, despite increased risk of AD.

Exclusion Criteria:

Individuals who meet any of the following criteria will be excluded from participation in this study:

I. Below age 50
II. Documentation of prior dementia diagnosis

Exclusion criteria are justified because those below age 50 and those with dementia are ineligible for the existing APT Webstudy.

4. Recruitment/Screening Process (sampling strategy)

Our study partner, CCRMC, will provide the research team with an anonymous dataset that contains a unique code for all CCRMC patients who meet the inclusion and exclusion criteria for the study as well as their age in years, gender, race/ethnicity, preferred communication language (English/Spanish), preferred mode of communication from the health system (SMS or email) and insurance type. Only the health system, CCRMC, will be able to ascertain the identity of patients from these codes. The research team will not have access to the codes at any time. This anonymous dataset will be used for the purposes of randomly assigning patients to one of the three study arms: (1) message-only arm, (2) small financial incentive arm or (3) prize opportunity arm. Randomization will be stratified 1:1:1 by age group (50-64, 65 and over), race and ethnic status (Latino/Hispanic, non-Hispanic White, non-Hispanic Black, non-Hispanic Asian, Other) and message modality (email or text). The other covariates in the dataset will be used to check for balance in observable characteristics across study arm. We have worked with CCRMC on several prior projects and have an established process for collaboration.

The study team will then return the anonymous data (with a unique code only known to the health system) to CCRMC with a new study arm variable showing the assigned study arm for each of the included patients. The health system will then send SMS messages or e-mail, depending on the member's preferred mode of communication *(*SMS/e-mail), inviting members to participate in the APT Webstudy and offering them an incentive (small certain or prize opportunity) if the member was assigned to one of the incentive arms. All messages will be *sent by the health system and* delivered in English or Spanish, depending on the preferred language. After an initial small pilot test of the messaging, remaining eligible members will be messaged in sequential waves. The pilot will be deployed to about 500 eligible members and will only include the message arm and the small incentive arm.

For this recruitment study, there is no explicit consent process for study participants. Only those health system members who have not opted out of communications from CCRMC will be messaged. By design, 1/3 of eligible members will be offered $25 for enrolling in the APT Webstudy and 1/3 will be entered into a drawing for a $2500 prize. Importantly, any patient who chooses to enroll in the APT Webstudy will be consented in that study directly during the registration and enrollment process.

5. Methods

All CCRMC patients who meet the inclusion criteria will receive the same basic email or SMS message, inviting them to participate in the APT Webstudy. Eligible CCRMC patients will be allocated 1:1:1 to 3 study arms. The first arm, the messaging only arm, will just receive the invite message. The 2nd arm will receive, along with the invite, the offer of a $25 gift card for completing enrollment into the APT Webstudy. The 3rd arm will receive, along with the invite, an offer of entry into a $2500 prize drawing for completing enrollment into the APT Webstudy. The prize opportunity will be designed to allow participants to have a 1 in 100 chance of winning a $2500 prize, thus the prize opportunity has the same expected value per enrollee as the certain $25 incentive. Registration for and enrollment in the APT Webstudy includes web-based capture of demographic, medical, lifestyle and risk factors, as well as longitudinal web-based cognitive testing and symptom questionnaires. One cognitive assessment, the Cogstate Brief Battery, is a cognitive performance test; the other, the Cognitive Function Index (CFI), is a series of 15 questions probing subjective concerns related to cognitive performance. Participants will qualify as enrolled in the APT Webstudy if they complete the registration process as well as at least one of the two cognitive assessments. None of the data captured in the APT Webstudy data will be shared with the research team.

Invitations will be made via SMS or e-mail, depending on members' previously indicated mode of communication with the health system.

The intervention will be implemented by our collaborating partner, Contra Costa Health Services (CCHS), which runs Contra Costa Regional Medical Center (CCRMC) The trial team will track returned/invalid email or phone numbers as well as requests to opt out of communications at the participant level.

Randomization will be developed by the trial statistical team and shared with CCHS for implementation. Randomization will be stratified by age group (50-64, 65 and over), race/ethnic status (Latino/Hispanic, non-Hispanic White, non-Hispanic Black, non-Hispanic Asian, Other) and message modality (email or text) and will use a 1:1:1 allocation ratio across the three intervention arms. This is a single-blind study; only the intervention team and the statistical team will be unblinded to the intervention.

Primary Endpoint: APT Webstudy enrollment rate based on completion of at least one of the two APT Webstudy cognitive assessments (Cognitive Function Instrument [CFI] and/or Cogstate Brief Battery). Enrollment will be captured by the APT Webstudy team, which is housed at ATRI, and will be shared with this study using the unique, anonymous code provided by CCRMC.

Secondary Endpoints: 1) Cost per enrollee and 2) Enrollees' rate of completion of both APT Webstudy cognitive assessments (CFI and Cogstate Brief Battery). Cost data are based on incentive payment costs while completion rate of both cognitive assessments will come from the APT Webstudy team and will be shared along with the unique, anonymous code provided by CCRMC.

Flow Diagram

Pre-Screening by Staff at Contra Costa Health Services (CCHS)

List of individuals eligible to receive invitation to APT Webstudy

Time 1: Invitations sent out by e-mail or SMS

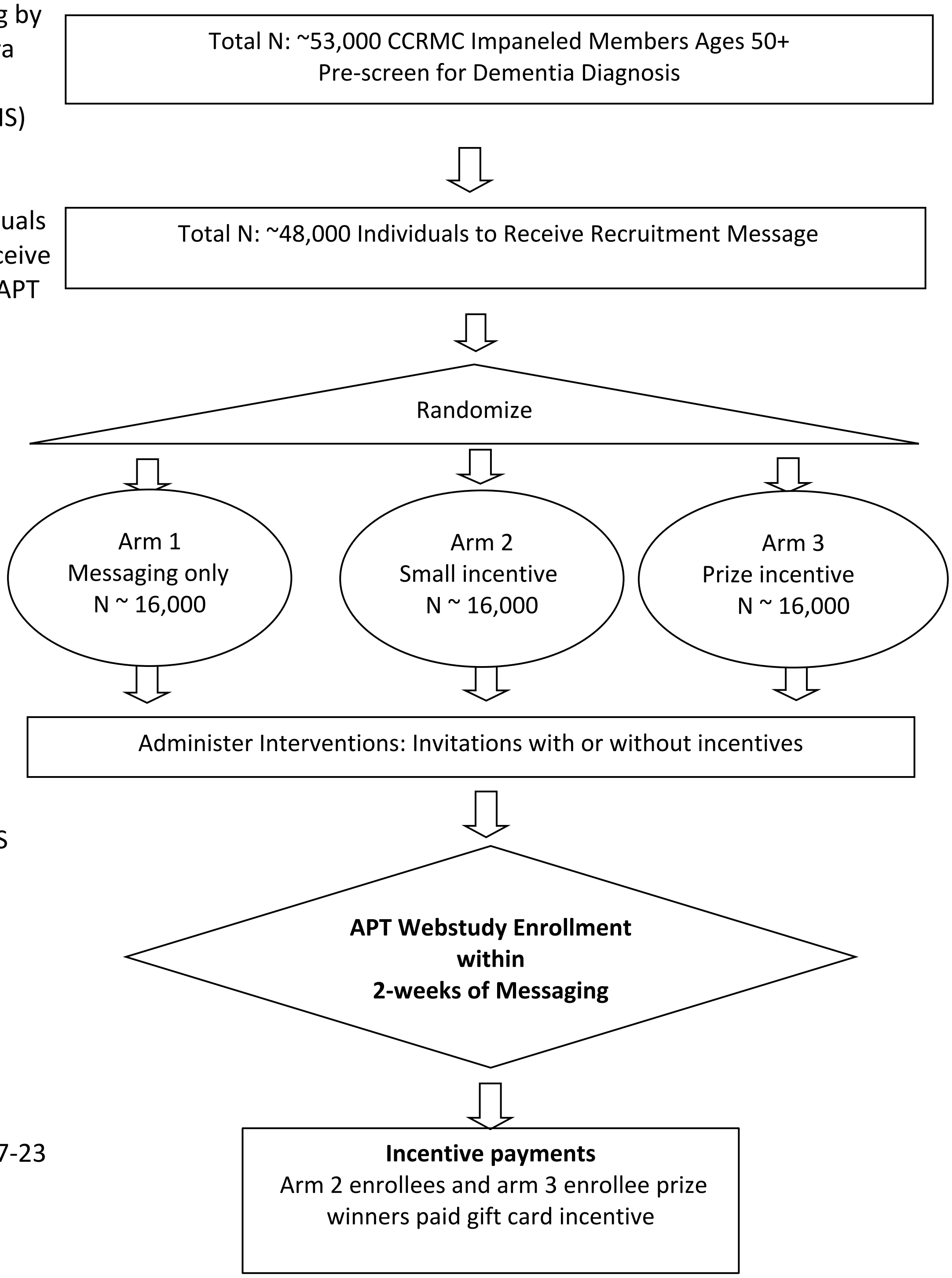

Revised 03-07-23

a. Data and Hypotheses

Our final dataset will include the full sample of people we attempted to message, i.e., the anonymous dataset that we used for randomization along with the outcome measures from ATRI on APT Webstudy enrollment, incentive payments, and completion of both cognitive assessments.

Below we provide our main null hypotheses:

Aim 1, Primary Endpoint: Enrollment Rates

$H1_{null}$: Financial incentives, certain small financial incentive or a prize opportunity incentive, will result in similar enrollment rates of CCRMC patients into the APT Webstudy relative to a message-only arm (active control).

Aim 2, $H2_{null}$: A prize opportunity incentive will result in similar enrollment rate of CCRMC patients into the APT Webstudy as a certain small financial incentive with the same expected value.

- Secondary Endpoint(s): Cost per enrollee

$H2_{null}$: The cost per APT Webstudy enrollee will be similar for the financial incentive arms compared with the message-only arm.

- Secondary Endpoint(s): Completion rates for both cognitive assessments

$H3_{null}$: Completion rates for both cognitive assessments will be similar for the financial incentive arms compared with the message-only arm.

b. Sample size determination

Data used in the power analysis are based on members ages 50 and over without MCI/Dementia currently included in the CCHS database. We power the study for the primary endpoint – enrollment rates. We based our power calculations on a two-sided chi-square test for detecting a difference between two proportions, assuming a type I error rate of 2.5% (to account for the two hypotheses of interest). With a sample size of 16,000 members in each treatment arm (48,000 overall), and assuming an enrollment rate of 2% in the active control arm, we have 90% power to detect an absolute difference of 0.59% or higher (an enrollment rate of 2.59% or higher) in either of the two financial incentive groups.

We also plan to conduct sub-group analyses, with a particular focus on racial and ethnic minorities. With sample sizes of 2100, 3600, 2940, and 5380 per arm in the Black, Asian, Latino(a) and White sub-groups, and assuming the same 2% enrollment rate in the active control arm, we have 90% power to detect an enrollment rate of 1.8%, 1.34%, 1.5% and 1.07% respectively in either of the two financial incentive arms. The sample size also provides adequate power to detect meaningful differences in other pre-specified populations of interest (based on primary language (English/Spanish), and age group (50-64,

65-74, 75 and over).

c. Statistical Analysis

The primary analysis will be based on the intention-to-treat (ITT) population of all messaged patients who are empaneled at Contra Costa Regional Medical Center (CCRMC), who are ages 50 and older and have no prior documentation of dementia.

Participant demographics and characteristics at time of messaging will be summarized for each intervention arm, using frequencies and percentages for categorical variables, and using mean, standard deviation, and quartiles for continuous variables. Comparisons across the three intervention groups will be done using a Chi-square test for categorical variables and an analysis of variance (ANOVA) test for continuous variables with Fisher's Exact Test or Dunnett's post-hoc tests, for categorical and continuous data respectively, to compare each active intervention arm against the control arm.

Our primary outcome is the enrollment status at 2-weeks. A multivariable logistic regression analysis will be performed to study the association between enrollment status and intervention arms (financial incentive arms vs active control arm) adjusting for age group (50-64, 65 and over), race and ethnic status (Latino/Hispanic, non-Hispanic White, non-Hispanic Black, non-Hispanic Asian, Other) and message modality (email or text). Separate analyses will be conducted for each financial incentive intervention arm against the active control arm.

All results will be reported as point estimates (e.g., percentage difference between groups) and interval estimates (two-sided 95% confidence intervals). All hypothesis tests to test group differences between each of the active intervention arms and the active control arm will be conducted at a two-sided alpha level of 0.025.

Our secondary outcomes are cost per enrollee and completion of both cognitive assessments in the APT Webstudy. A multivariable linear regression will be performed to study the association between the cost per enrollee and intervention arms (financial incentive arm, active control arm) adjusting for age group (50-64, 65 and over), race and ethnic status (Latino/Hispanic, non-Hispanic White, non-Hispanic Black, non-Hispanic Asian, Other) and message modality (email or text). The other secondary outcome, completion of both cognitive assessments in the APT Webstudy, will be analyzed using a multivariable logistic regression analogous to the analysis of the primary outcome. Analyses of the secondary outcomes will also be conducted at a two-sided alpha level of 0.025, without further adjustment for multiplicity.

Prespecified sub-group analyses based on age group (50-64, 65and over), race/ethnicity (Latino/Hispanic, non-Hispanic White, non-Hispanic Black, non-Hispanic Asian, Other) and message modality (email or text) will be conducted on all primary and secondary outcomes using analogous methods to the overall analysis. In addition, if feasible with available sample size, we will analyze sub-groups at the intersection of two or more of the identities of interest, e.g., racial/ethnic groups by age-group.

d. Data Protection, Storage and Sharing

Contra Costa Health Services (CCHS), which runs Contra Costa Regional Medical Center, will conduct an initial screen of their EMR and provide the research team with a de-identified dataset with basic demographics (age, sex, race/ethnicity and primary language) of patients ages 50 and above without a dementia diagnosis. That dataset will be used to randomize patients to study arms. A partial waiver of HIPAA authorization will be requested from IRB for the purposes of identifying qualified individuals. Individuals recruited to the APT study will be consented within the APT Webstudy system.

Data shared for randomization will be de-identified. Messaging will be performed by the health system. Deidentified data will be transferred between Contra Costa Health System (CCHS) and USC for the purposes of randomization. USC will also provide CCHS with the unique deidentified codes for members who enroll in the APT Webstudy. Only CCHS holds the key to identify these members. Where applicable the health system will make incentive payments to these members. All data transfers between CCHS and USC will rely on the USC secure file sharing platform, Box, which encrypts data during transfer and at rest. USC and CCHS will execute appropriate data use agreement(s) before transferring any data.

Measures Taken to Ensure Confidentiality of Data Shared per the AHA Data Sharing Policies

It is AHA policy that the results and accomplishments of the activities that it funds should be made available to the public (see *https://professional.heart.org/en/research-programs/aha-research-policies-and-awardee-hub/open-science-policy-statements-for-aha-funded-research*). The PIs will ensure all mechanisms used to share data will include proper plans and safeguards for the protection of privacy, confidentiality, and security for data dissemination and reuse (e.g., all data will be thoroughly de-identified and will not be traceable to a specific study participant). Plans for archiving and long-term preservation of the data will be implemented, as appropriate.

Participant privacy and confidentiality for the APT Webstudy will be protected as described in USC IRB #HS-17-00746. No added risks to privacy or confidentiality are anticipated. All user and study data are stored and maintained on servers hosted on Amazon Web Services under an Enterprise Agreement and a Business Associate Agreement with USC which stipulates rights and responsibilities between both parties. All data is encrypted in transit and at rest.

Data collected for this study will be analyzed and stored at the University of Southern California (USC). APT Webstudy enrollment data will be maintained at USC's Alzheimer's Therapeutic Research Institute (ATRI) and data usage will be guided by the APT Webstudy data sharing plan.

References

1. Mayeda, E.R., et al., *Inequalities in dementia incidence between six racial and ethnic groups over 14 years.* Alzheimers Dement, 2016. **12**(3): p. 216-24.

2. Mehta, K.M. and G.W. Yeo, *Systematic review of dementia prevalence and incidence in United States race/ethnic populations.* Alzheimers Dement, 2017. **13**(1): p. 72-83.

3. Alzheimer's Association, *Special Report Race, Ethnicity and Alzheimer's In America*. 2021: Chicago.

4. Lennon, J.C., et al., *Black and White individuals differ in dementia prevalence, risk factors, and symptomatic presentation.* Alzheimers Dement, 2021.

5. Filshtein, T.J., et al., *Neuropathological Diagnoses of Demented Hispanic, Black, and Non-Hispanic White Decedents Seen at an Alzheimer's Disease Center.* J Alzheimers Dis, 2019. **68**(1): p. 145-158.

6. Manly, J.J., et al., *Effect of literacy on neuropsychological test performance in nondemented, education-matched elders.* J Int Neuropsychol Soc, 1999. **5**(3): p. 191-202.

7. U.S. Census, *2020 U.S. Population More Racially and Ethnically Diversed Than Measured in 2010.* 2021.

8. Sharrocks, K., et al., *The impact of socioeconomic status on access to cancer clinical trials.* Br J Cancer, 2014. **111**(9): p. 1684-7.

9. Canevelli, M., et al., *Socioeconomic disparities in clinical trials on Alzheimer's disease: a systematic review.* Eur J Neurol, 2018. **25**(4): p. 626-e43.

10. U.S. Food and Drug Administration Office of Woment's Health, *Dialogues on Diversifying Clinical Trials*. 2011: Washington, D.C.

11. U.S. Food & Drug Administration, *Enhancing the Diversity of Clinical Trial Populations — Eligibility Criteria, Enrollment Practices, and Trial Designs Guidance for Industry.* Guidance Document, 2020.

12. Aging, N.I.o., *Together We Make the Difference: National Strategy for Recruitment and Participation in Alzheimer's and Related Dementials Clincial Research.* 2018.

13. Gilmore-Bykovskyi, A.L., et al., *Recruitment and retention of underrepresented populations in Alzheimer's disease research: A systematic review.* Alzheimers Dement (N Y), 2019. **5**: p. 751-770.

14. Zhou, Y., et al., *African Americans are less likely to enroll in preclinical Alzheimer's disease clinical trials.* Alzheimers Dement (N Y), 2017. **3**(1): p. 57-64.

15. Aisen, P.S., et al., *The Trial-Ready Cohort for Preclinical/Prodromal Alzheimer's Disease (TRC-PAD) Project: An Overview.* J Prev Alzheimers Dis, 2020. **7**(4): p. 208-212.

16. Watson, J.L., et al., *Obstacles and opportunities in Alzheimer's clinical trial recruitment.* Health Aff (Millwood), 2014. **33**(4): p. 574-9.

17. Chen, Y., et al., *Analysis of dementia in the US population using Medicare claims: Insights from linked survey and administrative claims data.* Alzheimers Dement (N Y), 2019. **5**: p. 197-207.

18. Thunell, J., P. Ferido, and J. Zissimopoulos, *Measuring Alzheimer's Disease and Other Dementias in Diverse Populations Using Medicare Claims Data.* J Alzheimers Dis, 2019. **72**(1): p. 29-33.

19. Zhu, Y., et al., *Sex, Race, and Age Differences in Prevalence of Dementia in Medicare Claims and Survey Data.* J Gerontol B Psychol Sci Soc Sci, 2021. **76**(3): p. 596-606.

20. Robinson, K.A., et al., *Systematic review identifies number of strategies important for retaining study participants.* J Clin Epidemiol, 2007. **60**(8): p. 757-65.

21. Robinson, K.A., et al., *Updated systematic review identifies substantial number of retention strategies: using more strategies retains more study participants.* J Clin Epidemiol, 2015. **68**(12): p. 1481-7.

22. Kakumanu, S., et al., *Cost analysis and efficacy of recruitment strategies used in a large pragmatic community-based clinical trial targeting low-income seniors: a comparative descriptive analysis.* Trials, 2019. **20**(1): p. 577.

23. Huynh, L., et al., *Cost-effectiveness of health research study participant recruitment strategies: a systematic review.* Clin Trials, 2014. **11**(5): p. 576-83.

24. Dilworth-Anderson, P. and M.D. Cohen, *Beyond diversity to inclusion: recruitment and retention of diverse groups in Alzheimer research.* Alzheimer Dis Assoc Disord, 2010. **24 Suppl**: p. S14-18.

25. Santoyo-Olsson, J., et al., *An innovative multiphased strategy to recruit underserved adults into a randomized trial of a community-based diabetes risk reduction program.* Gerontologist, 2011. **51 Suppl 1**: p. S82-93.

26. Huang, B., et al., *Strategies for recruitment and retention of underrepresented populations with chronic obstructive pulmonary disease for a clinical trial.* Bmc Medical Research Methodology, 2019. **19**.

27. Kogan, J.N., et al., *Increasing minority research participation through collaboration with community outpatient clinics: the STEP-BD Community Partners Experience.* Clinical Trials, 2009. **6**(4): p. 344-354.

28. Barankay, I., et al., *Effect of Patient Financial Incentives on Statin Adherence and Lipid Control: A Randomized Clinical Trial.* JAMA Netw Open, 2020. **3**(10): p. e2019429.

29. Mitchell, M.S., et al., *Financial incentives for exercise adherence in adults: systematic review and meta-analysis.* Am J Prev Med, 2013. **45**(5): p. 658-67.

30. Parkinson, B., et al., *Designing and using incentives to support recruitment and retention in clinical trials: a scoping review and a checklist for design.* Trials, 2019. **20**(1): p. 624.

31. Zheng, L., et al., *Financial incentives for hypertension control: rationale and study design.* Trials, 2020. **21**(1): p. 134.

32. Vijayaraghavan, M., et al., *Racial/Ethnic Differences in the Response to Incentives for Quitline Engagement.* Am J Prev Med, 2018. **55**(6 Suppl 2): p. S186-S195.

33. Lasser, K.E., et al., *Effect of Patient Navigation and Financial Incentives on Smoking Cessation Among Primary Care Patients at an Urban Safety-Net Hospital: A Randomized Clinical Trial.* JAMA Intern Med, 2017. **177**(12): p. 1798-1807.

34. Law, A.C., et al., *Lottery-Based Incentives and COVID-19 Vaccination Rates in the US.* JAMA Intern Med, 2022.
