# Supplementary material for: Financial Incentives to Increase Diversity of Older Participants in a Memory Concerns Registry: A Randomized Clinical Trial
Source: JAMA Health Forum. 2025 Aug 22;6(8):e252273. doi: 10.1001/jamahealthforum.2025.2273 (PMC12374222; doi:10.1001/jamahealthforum.2025.2273)
Supplement: Supplement 2. — eAppendix 1. Study Overview eAppendix 2. Study Messages eFigure. Odds Ratios From Models Estimated by Subgroups eTable 1. Odds Ratios for APT Webstudy Enrollment Estimated Relative to the Small Incentive Arm eTable 2. Odds Ratio for Enrollment in the APT Webstudy by Age Group, Sex, and Primary Insurance eTable 3. Odds Ratio for Completion of Both Screeners Overall and by Subgroup eTable 4. Marginal Cost Per Additional Enrollee eTable 5. Characteristics of APT Webstudy Enrollees Overall and by Arm [file jamahealthforum-e252273-s002.pdf]

Supplemental Online Content

Jacobson M, Molina-Henry D, Chang TY, et al. Financial incentives to increase diversity of older participants in a memory concerns registry: a randomized clinical trial. *JAMA Health Forum.* 2025;6(8):e252273. doi:10.1001/jamahealthforum.2025.2273

eAppendix 1. Study Overview
eAppendix 2. Study Messages
eFigure. Odds Ratios From Models Estimated by Subgroups
eTable 1. Odds Ratios for APT Webstudy Enrollment Estimated Relative to the Small Incentive Arm
eTable 2. Odds Ratio for Enrollment in the APT Webstudy by Age Group, Sex, and Primary Insurance
eTable 3. Odds Ratio for Completion of Both Screeners Overall and by Subgroup
eTable 4. Marginal Cost Per Additional Enrollee
eTable 5. Characteristics of APT Webstudy Enrollees Overall and by Arm

This supplementary material has been provided by the authors to give readers additional information about their work.

© 2025 Jacobson M et al. *JAMA Health Forum*.

eAppendix 1. Study Overview

Ethics Approval

Our protocol was approved by the Institutional Review Board (IRB) at the University of Southern California (approval number: UP-22-01037) and Contra Costa Health Service's Institutional Review Committee.

Data collection and analysis was pre-registered at clinicaltrials.gov (NCT06300242) https://clinicaltrials.gov/study/NCT06033066. Detailed discussion of the pre-analysis plan and data analysis is in the Supplemental Materials (SM). The purpose of the project was to study the impact of financial incentives on enrollment into the APT Webstudy, a brain health registry aimed at accelerating clinical trials for Alzheimer's Disease therapeutics.

All code for reproducing the tables and figures in the manuscript and supplemental materials will be posted on OSF prior to publication. We cannot publicly post individual-level data. Data containing individual-level health information are typically not made publicly available to protect patient privacy. However, data can be made available upon request. See the Data Sharing Statement in Supplement 3.

Details on the RCT

Overview

Our pre-registered RCT was designed to assess the impact of offering financial incentives (a certain small financial incentive or a randomized prize incentive) on enrollment rates of a population of diverse low-income adults ages 50 years and older into the APT Webstudy.

Sample and Design

Study participants were patients empaneled at Contra Costa Regional Medical Center (CCRMC) who, were aged 50 and over, had no documentation of dementia in their electronic medical record and had not opted out of receiving health system messages. These patients were randomly allocated to one of three conditions: sent an invitation message with the offer of a $25 incentive for enrolling in the APT Webstudy, sent an invitation message with the offer of entry into a prize drawing with a $2500 award for enrolling in the APT Webstudy, sent an invitation message without an incentive (active control group). Messages were sent in English and Spanish only, since the APT Webstudy is only available in these languages.

© 2025 Jacobson M et al. *JAMA Health Forum*.

eAppendix 2. Study Messages

Basic Messages

1a. Basic Message – English

Subject: You are invited to participate in a brain health program

[First Name]

Contra Costa Health Services invites you to participate in an online research study to monitor brain health, the Alzheimer Prevention Trials (APT) Webstudy. By participating you can learn more about your brain health and help researchers learn more about how we can prevent dementias that cause severe memory loss, like Alzheimer's disease dementia. You can join the Webstudy from the comfort of your home by completing a 5 min registration and two brief cognitive tests.

This invitation is intended only for you as a member of Contra Costa Health Services, and should not be shared with others. If you have any questions regarding this invitation, please email us at info@aptwebstudy.org, or call us at 1-858-877-3135.

To sign up click here: [INSERT LINK HERE]

1b. Basic Message – Spanish

Subject: Usted está invitado a participar en un programa de salud cerebral

[First Name]:

Los Servicios de Salud de Contra Costa le invitan a participar en un estudio de investigación para monitorear la salud cerebral, que se lleva acabo en línea, el Estudio Web para la Prevención del Alzheimer. Al participar, puede aprender más sobre la salud de su cerebro y ayudar a los investigadores a aprender más sobre cómo podemos prevenir las demencias que causan pérdida grave de memoria, como la demencia de la enfermedad de Alzheimer. Puede participar en el Estudio Web desde la comodidad de su hogar completando un registro de 5 minutos y dos breves pruebas cognitivas.

Esta invitación está destinada únicamente a usted como paciente de Los Servicios de Salud de Contra Costa , y no debe compartirse con otros.

Si tiene alguna pregunta sobre esta invitación, envíenos un correo electrónico a info@aptwebstudy.org, o llámenos al 1-858-877-3135.

Para inscribirse, haga clic aquí: [INSERT LINK HERE]

© 2025 Jacobson M et al. *JAMA Health Forum*.

2a. $25 incentive arm Message

Subject: You are invited to participate in a brain health program.

[First Name]

Contra Costa Health Services invites you to participate in an online research study to monitor brain health, the Alzheimer Prevention Trials (APT)l Webstudy. By participating you can learn more about your brain health and help researchers learn more about how we can prevent dementias that cause severe memory loss, like Alzheimer's disease dementia. You can join the APT Webstudy from the comfort of your home by completing a 5 min registration and brief two cognitive tests.

If you sign up and complete the first memory test within 7 days, you will receive a 25 USD Amazon reward.

This invitation is intended only for you as a member of Contra Costa Health Services, and should not be shared with others. If you have any questions regarding this invitation, please email us at info@aptwebstudy.org, or call us at 1-858-877-3135.

To sign up click here: [INSERT LINK HERE]

2b. $25 incentive arm Message Spanish

Subject: Usted está invitado a participar en un programa de salud cerebral

[First Name]:

Los Servicios de Salud de Contra Costa le invita a participar en un estudio de investigación en línea para monitorear la salud del cerebro, Estudio Web para la Prevención del Alzheimer. Al participar, puede aprender más sobre la salud de su cerebro y ayudar a los investigadores a aprender más sobre cómo podemos prevenir las demencias que causan pérdida grave de memoria, como la demencia de la enfermedad de Alzheimer. Puede participar en el Estudio Web para la Prevención del Alzheimer desde la comodidad de su hogar completando un registro de 5 minutos y dos breves pruebas cognitivas.

Si se inscribe y completa la primera prueba de memoria dentro de 7 días, recibirá una recompensa de Amazon de 25 dólares.

Esta invitación está destinada únicamente a usted como paciente de Los Servicios de Salud de Contra Costa , y no debe compartirse con otros.

Si tiene alguna pregunta sobre esta invitación, envíenos un correo electrónico a info@aptwebstudy.org, o llámenos al 1-858-877-3135.
Para inscribirse, haga clic aquí: [INSERT LINK HERE]

© 2025 Jacobson M et al. *JAMA Health Forum*.

3a. $2500 prize incentive arm message

Subject: You are invited to participate in a brain health program.

[First Name]

Contra Costa Health Services invites you to participate in an online research study to monitor brain health, the Alzheimer Prevention Trials (APT)l Webstudy. By participating you can learn more about your brain health and help researchers learn more about how we can prevent dementias that cause severe memory loss, like Alzheimer's disease dementia. You can join the APT Webstudy from the comfort of your home by

If you sign up and complete the first memory test within 7 days, you will be entered into a drawing for a 2500 USD Amazon reward. The selected winners will be notified within 90 days.

This invitation is intended only for you as a member of Contra Costa Health Services, and should not be shared with others. If you have any questions regarding this invitation, please email us at info@aptwebstudy.org, or call us at 1-858-877-3135.

To sign up click here: [INSERT LINK HERE]

3b. $2500 prize incentive arm message (Spanish)

Subject: Usted está invitado a participar en un programa de salud cerebral

[First Name]:

Los Servicios de Salud de Contra Costa le invita a participar en un estudio de investigación en línea para monitorear la salud del cerebro, Estudio Web para la Prevención del Alzheimer. Al participar, puede aprender más sobre la salud de su cerebro y ayudar a los investigadores a aprender más sobre cómo podemos prevenir las demencias que causan pérdida grave de memoria, como la demencia de la enfermedad de Alzheimer. Puede participar en el Estudio Web para la Prevención del Alzheimer desde la comodidad de su hogar completando un registro de 5 minutos y dos breves pruebas cognitivas.

Si se inscribe y completa la primera prueba de memoria dentro de 7 días, entrarás en un sorteo de una recompensa de Amazon de 2500 dólares. Los ganadores seleccionados serán notificados en un plazo de 90 días.

Esta invitación está destinada únicamente a usted como paciente de Los Servicios de Salud de Contra Costa , y no debe compartirse con otros.

Si tiene alguna pregunta sobre esta invitación, envíenos un correo electrónico a info@aptwebstudy.org, o llámenos al 1-858-877-3135.
Para inscribirse, haga clic aquí: [INSERT LINK HERE

© 2025 Jacobson M et al. *JAMA Health Forum*.

eFigure. Odds Ratios From Models Estimated by Subgroups

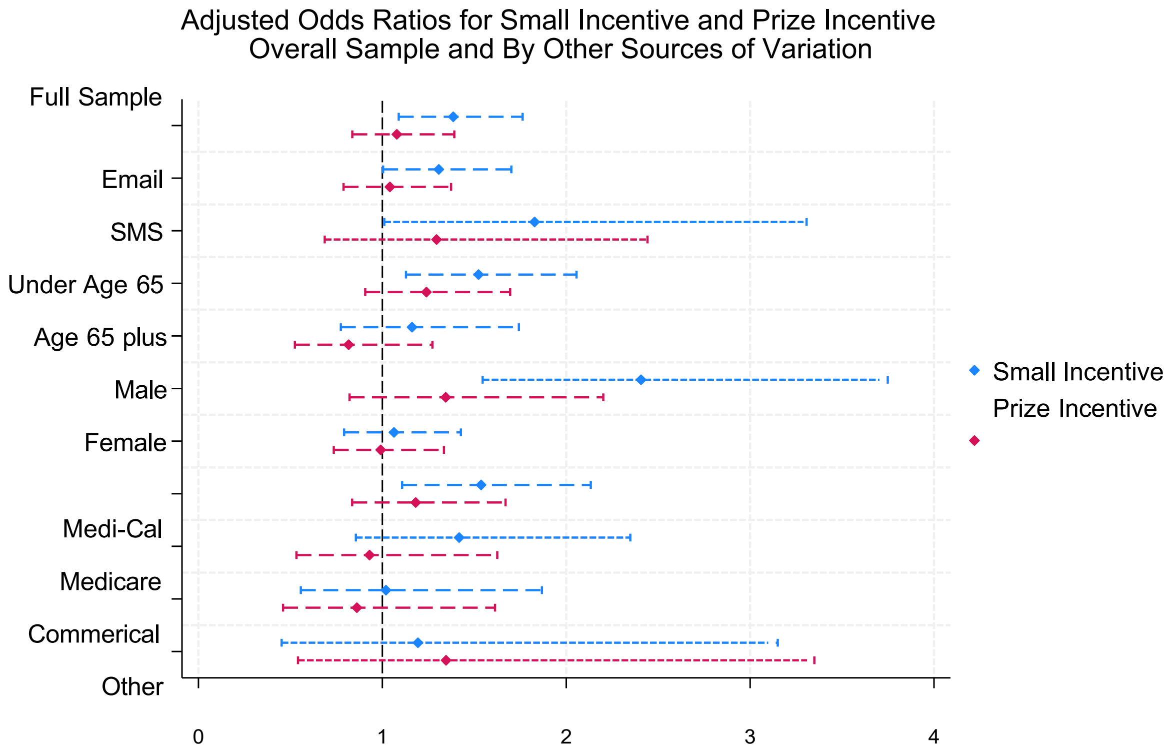

© 2025 Jacobson M et al. *JAMA Health Forum*.

eTable 1. Odds Ratios for APT Webstudy Enrollment Estimated Relative to the Small Incentive Arm[a]

	Enrollment rate (%)[a]	Unadjusted[b] Odds Ratio	95% CI	P value[c]	Adjusted Odds Ratio	95% CI[d]	P value[c]
All patients[b]	1.07%						
Message Only		0.723	[0.57, 0.92]	0.008	0.722	[0.57, 0.92]	0.008
Prize Incentive		0.780	[0.62, 0.99]	0.038	0.779	[0.62, 0.99]	0.037
Ages 50-64[b]	1.33%						
Message Only		0.658	[0.49, 0.80]	0.006	0.657	[0.40, 0.89]	0.006
Prize Incentive		0.815	[0.62, 1.08]	0.154	0.814	[0.61, 1.08]	0.153
Ages >=65[b]	0.75%						
Message Only		0.862	[0.58, 1.29]	0.472	0.861	[0.57, 1.29]	0.470
Prize Incentive		0.704	[0.46, 1.08]	0.109	0.703	[0.46, 1.08]	0.108
Email Message[b]	1.42%						
Message Only		0.765	[0.59, 1.00]	0.046	0.764	[0.59, 1.00]	0.046
Prize Incentive		0.796	[0.61, 1.03]	0.087	0.796	[0.61, 1.03]	0.086
SMS Message[b]	0.53%						
Message Only		0.547	[0.30, 0.99]	0.046	0.547	[0.30, 0.99]	0.046
Prize Incentive		0.709	[0.41, 1.23]	0.218	0.708	[0.41, 1.23]	0.217
Asian[b]	0.47%						
Message Only		0.532	[0.23, 1.26]	0.151	0.532	[0.23, 1.26]	0.150
Prize Incentive		0.732	[0.34, 1.60]	0.434	0.732	[0.34, 1.60]	0.433
Black[b]	0.84%						
Message Only		0.942	[0.48, 1.87]	0.865	0.942	[0.47, 1.87]	0.865
Prize Incentive		0.883	[0.44, 1.77]	0.727	0.884	[0.44, 1.78]	0.729

© 2025 Jacobson M et al. *JAMA Health Forum*.

Hispanic[b]	0.63%						
Message Only		1.000	[0.57, 1.77]	1.000	1.000	[0.57, 1.77]	1.000
Prize Incentive		0.832	[0.46, 1.51]	0.545	0.832	[0.46, 1.51]	0.545
White[b]	2.20%						
Message Only		0.624	[0.45, 0.87]	0.006	0.623	[0.45, 0.87]	0.006
Prize Incentive		0.681	[0.49, 0.95]	0.022	0.680	[0.49, 0.95]	0.022
Other[b]	0.77%						
Message Only		0.799	[0.37, 1.71]	0.563	0.798	[0.37, 1.71]	0.562
Prize Incentive		1.201	[0.60, 2.39]	0.602	1.203	[0.60, 2.40]	0.600

[a] The excluded arm in these models is the small incentive arm.
[b] Each panel "All participants," "Asian," … reports the Small and Prize incentive arm estimates from separate logistic regression models.
[c] P values are from a test of the null that the odds of enrollment in a given arm (Small Incentive or Prize Incentive) is the same as the odds in the Message-only (active control) arm.
[d] Models for all participants adjust for race and ethnicity (Asian, Black, Hispanic, or Other relative to White), age group (age 65 and over relative to under 65) and message modality (email relative to text). Models by sub-group only control for other strata variables. For example, models by race control for age group and message modality

© 2025 Jacobson M et al. *JAMA Health Forum*.

eTable 2. Odds Ratio for Enrollment in the APT Webstudy by Age Group, Sex, and Primary Insurance

	Enrollment rate (%)[a]	Unadjusted Odds Ratio	95% CI	P value[b]	Adjusted[c] Odds Ratio	95% CI	P value[b]
Ages 50-64[a]	0.88%						
Small Incentive		1.52	[1.1, 2.1]	0.006	1.523	[1.1, 2.1]	0.006
Prize Incentive		1.238	[0.9, 1.7]	0.179	1.24	[0.9, 1.7]	0.178
Ages >=65 [a]	0.65%						
Small Incentive		1.16	[0.8, 1.7]	0.472	1.161	[0.8, 1.7]	0.47
Prize Incentive		0.817	[0.5, 1.3]	0.37	0.817	[0.5, 1.3]	0.371
Email Message [a]	1.09%						
Small Incentive		1.307	[1.0, 1.7]	0.046	1.308	[1.0, 1.7]	0.046
Prize Incentive		1.041	[0.8, 1.4]	0.777	1.041	[0.8, 1.4]	0.776
SMS Message [a]	0.29%						
Small Incentive		1.827	[1.0, 3.3]	0.046	1.83	[1.0, 3.3]	0.046
Prize Incentive		1.295	[0.7, 2.4]	0.425	1.295	[0.7, 2.4]	0.425
Male [a]	0.43%						
Small Incentive		2.375	[1.53, 3.7]	<0.001	2.407	[1.55, 3.75]	<0.001
Prize Incentive		1.335	[0.82, 2.18]	0.250	1.345	[0.82, 2.20]	0.239
Female [a]	1.04%						
Small Incentive		1.068	[0.80, 1.43]	0.661	1.063	[0.792, 1.427]	0.683
Prize Incentive		0.993	[0.74, 1.34]	0.964	0.991	[0.736, 1.335]	0.953
Medi-Cal [a]	0.72%						
Small Incentive		1.528	[1.10, 2.12]	0.011	1.537	[1.11, 2.13]	0.010
Prize Incentive		1.183	[0.84, 1.67]	0.340	1.181	[0.836, 1.67]	0.345
Medicare [a]	0.62%						
Small Incentive		1.411	[0.853, 2.33]	0.180	1.418	[0.856, 2.35]	0.175

© 2025 Jacobson M et al. *JAMA Health Forum*.

Prize Incentive		0.932	[0.534, 1.63]	0.803	0.930	[0.533, 1.625]	0.799
Commercial [a]	2.38%						
Small Incentive		1.046	[0.58, 1.90]	0.882	1.020	[0.557, 1.89]	0.950
Prize Incentive		0.886	[0.48, 1.65]	0.703	0.861	[0.460, 1.61]	0.641
Other Insurance [a]	0.53%						
Small Incentive		1.174	[0.452, 3.05]	0.742	1.194	[0.452, 3.15]	0.721
Prize Incentive		1.328	[0.533, 3.31]	0.543	1.346	[0.541, 3.35]	0.522

[a] Each panel “Ages 50-64,” “Ages>=65,” … reports the Small and Prize incentive arm estimates from separate logistic regression models.

[b] P values are from a test of the null that the odds of enrollment in a given arm (Small Incentive or Prize Incentive) is the same as the odds in the Message-only (active control) arm.

[c] Models adjust for race and ethnicity (Asian, Black, Hispanic, or Other relative to White), age group (age 65 and over relative to under 65) and message modality (email relative to text). Models by racial and ethnic sub-group only control for age group and message modality whereas models by age group adjust for race and ethnicity and message modality and models by message modality adjust for race and ethnicity and age group.

© 2025 Jacobson M et al. *JAMA Health Forum*.

eTable 3. Odds Ratio for Completion of Both Screeners Overall and by Subgroup

	Enrollment rate (%)[a]	Unadjusted[b] Odds Ratio	95% CI	P value	Adjusted[c] Odds Ratio	95% CI	P value
All patients	0.21%						
Small Incentive		1.815	[1.2, 2.8]	0.007	1.818	[1.2, 2.8]	0.007
Prize Incentive		1.062	[0.7, 1.7]	0.806	1.063	[0.7, 1.7]	0.805
Asian	0.03%						
Small Incentive		7.009	[0.9, 57.0]	0.069	7.02	[0.9, 57.1]	0.068
Prize Incentive		3	[0.3, 28.9]	0.342	3	[0.3, 28.9]	0.341
Black	0.15%						
Small Incentive		1.332	[0.3, 6.0]	0.708	1.332	[0.3, 6.0]	0.708
Prize Incentive		1.335	[0.3, 6.0]	0.706	1.336	[0.3, 6.0]	0.705
Hispanic	0.13%						
Small Incentive		0.8	[0.2, 3.0]	0.739	0.8	[0.2, 3.0]	0.739
Prize Incentive		0.6	[0.1, 2.5]	0.484	0.599	[0.1, 2.5]	0.484
White	0.40%						
Small Incentive		2.451	[1.4, 4.4]	0.003	2.456	[1.4, 4.4]	0.003
Prize Incentive		1	[0.5, 2.0]	1	1	[0.5, 2.0]	0.999
Other	0.36%						
Small Incentive		0.571	[0.2, 2.0]	0.371	0.57	[0.2, 2.0]	0.371
Prize Incentive		1.143	[0.4, 3.2]	0.797	1.143	[0.4, 3.2]	0.797
Ages 50-64	0.22%						
Small Incentive		1.724	[1.0, 3.1]	0.066	1.727	[1.0, 3.1]	0.066
Prize Incentive		1.111	[0.6, 2.1]	0.746	1.112	[0.6, 2.1]	0.745
Ages >=65	0.21%						
Small Incentive		1.932	[1.0, 3.7]	0.046	1.936	[1.0, 3.7]	0.045
Prize Incentive		1	[0.5, 2.1]	1	1	[0.5, 2.1]	0.999

© 2025 Jacobson M et al. *JAMA Health Forum*.

Email Message	0.31%						
Small Incentive		1.826	[1.2, 2.9]	0.011	1.828	[1.2, 2.9]	0.011
Prize Incentive		1.179	[0.7, 2.0]	0.522	1.18	[0.7, 2.0]	0.521
SMS Message	0.07%						
Small Incentive		1.75	[0.5, 6.0]	0.372	1.751	[0.5, 6.0]	0.372
Prize Incentive		0.25	[0.0, 2.2]	0.215	0.25	[0.0, 2.2]	0.215
Male	0.12%						
Small Incentive		3.340	[1.52, 7.36]	0.003	3.407	[1.55, 7.49]	0.002
Prize Incentive		1.514	[0.618, 3.71]	0.364	1.537	[0.629, 3.75]	0.345
Female	1.04%						
Small Incentive		1.305	[0.765, 2.23]	0.328	1.300	[0.761, 2.22]	0.337
Prize Incentive		0.910	[0.51, 1.63]	0.750	0.911	[0.510, 1.63]	0.754
Medi-Cal	0.72%						
Small Incentive		3.358	[1.53, 7.40]	0.003	3.388	[1.537, 7.464]	0.002
Prize Incentive		1.999	[0.855, 4.67]	0.110	2.004	[0.856, 4.69]	0.109
Medicare	0.62%						
Small Incentive		1.371	[0.671, 2.80]	0.387	1.373	[0.671, 2.81]	0.386
Prize Incentive		0.543	[0.216, 1.36]	0.193	0.540	[0.216, 1.35]	0.189
Commercial	2.38%						
Small Incentive		1.046	[0.433, 2.53]	0.921	0.969	[0.401, 2.34]	0.944
Prize Incentive		0.615	[0.222, 1.70]	0.348	0.579	[0.211, 1.59]	0.289
Other Insurance	0.53%						
Small Incentive		3.133	[0.325,30.2]	0.323	3.061	[0.297,31.5	0.347
Prize Incentive		4.833	[0.564,41.4]	0.151	4.742	[0.557,40.4]	0.154

[a] Each panel "Ages 50-64," "Ages>=65," … reports the Small and Prize incentive arm estimates from separate logistic regression models.
[b] P values are from a test of the null that the odds of enrollment in a given arm (Small Incentive or Prize Incentive) is the same as the odds in the Message-only (active control) arm.

© 2025 Jacobson M et al. *JAMA Health Forum*.

[c] Models adjust for race and ethnicity (Asian, Black, Hispanic, or Other relative to White), age group (age 65 and over relative to under 65) and message modality (email relative to text). Models by racial and ethnic sub-group only control for age group and message modality whereas models by age group adjust for race and ethnicity and message modality and models by message modality adjust for race and ethnicity and age group.

© 2025 Jacobson M et al. *JAMA Health Forum*.

eTable 4. Marginal Cost Per Additional Enrollee

Study Arm	Enrollment (%)	Messaging Costs[a]	Messaging + Incentive Costs[b]
Message	0.78	$2.56	$2.56
Message + small incentive	1.07	$1.87	$26.87
Message + prize incentive	0.84	$2.38	$27.38

[a] Messaging costs assume a marginal cost of email of $0 and a marginal cost of texting of $0.05, which is taken from an industry blog. See https://simpletexting.com/blog/cost-of-mass-texting/ Since 40% of messages were sent by text, an average message cost 0.4 * $0.05 = $0.02. Thus, the marginal cost of an additional enrollee in the message only arm was $0.02 *100/0.78 or $2.56 per additional enrollee.
[b] Intervention arms cost an additional $25, in expectation, per enrollee. We add that to the marginal cost per additional enrollee in each of the intervention arms, accounting for differential enrollment per arm.

eTable 5. Characteristics of APT Webstudy Enrollees Overall and by Arm

	Overall (n=401)	Message (n=116)	Message + small incentive (n=160)	Message + prize incentive (n=125)
Race and ethnicity (%)				
Asian	8.48	6.9	9.38	8.8
Black	11.97	13.79	10.63	12
Hispanic	16.96	20.7	15	16
White	51.37	48.28	55.63	48.8
Other [a]	11.22	10.34	9.38	14.4
Female (%)	67.08	75.86	58.13	70.4
Age (mean, (SD))	61.4 (7.78)	62.42 (7.86)	60.98 (7.37)	61.15 (8.18)
Insurance (Primary)				
Medicaid	55.61	51.72	57.5	56.8
Medicare	21.7	22.41	23.13	19.2
Commercial	15.71	18.97	13.75	15.2
Other	6.98	6.9	5.63	8.8

[a] Other race includes participants who identified as American Indian/Alaska Native, Hawaiian/Pacific Islander, multi-racial, other race or declined to provide or were otherwise missing race information.

© 2025 Jacobson M et al. *JAMA Health Forum*.
