# Supplementary material for: Financial Incentives to Increase Diversity of Older Participants in a Memory Concerns Registry: A Randomized Clinical Trial
Source: JAMA Health Forum. 2025 Aug 22;6(8):e252273. doi: 10.1001/jamahealthforum.2025.2273 (PMC12374222; doi:10.1001/jamahealthforum.2025.2273)
Supplement: Supplement 3. — Data Sharing Statement [file jamahealthforum-e252273-s003.pdf]

Data Sharing Statement

Jacobson. Financial Incentives to Increase Diversity of Older Participants in a Memory Concerns Registry. *JAMA Health Forum*. Published August 22, 2025.
doi:10.1001/jamahealthforum.2025.2273

Data
Additional Information: NCT06033066
Data available: No

Additional Information
Explanation for why data not available: The experimental data analyzed in this paper were provided by Contra Costa Health Services and the APT Webstudy Team. We cannot publicly post individual-level data. Data containing individual-level health information are typically not made publicly available to protect patient privacy. Deidentified data from this work can be requested from Contra Costa Regional Medical Center through request to Rajiv Pramanik at Rajiv.Pramanik@cchealth.org and Tom Chang at tychang@usc.edu. All analytic programs and a file with summary data will be made available on OSF before publication: M.J. and T.Y.C, had deidentified access to all of the data in the study. These authors take responsibility for the integrity of the data and the accuracy of the data analysis.
